# GSA and AGHE: 75 Years of Leading Innovative, Interdisciplinary, Intergenerational, and Engaging Education

**DOI:** 10.1093/geroni/igaa057.1769

**Published:** 2020-12-16

**Authors:** Judith Howe, Marie Bernard

## Abstract

The AGHE Symposium will highlight the many ways that education has advanced interdisciplinary work, innovative programs, intergenerational services and community engagement in the field of aging over the last decades. Presenters will reflect on how these educational modalities can inform the future of gerontology education. Educators have innovated over decades to develop a field characterized by a diversity of programs, including internships, applied learning experiences in research, policy and practice, and leadership. The history of AGHE and interdisciplinary education are linked, with AGHE serving as an incubator for the advancement of change in this area. Over the course of the last five decades, funding from federal and foundation initiatives have resulted in multi-disciplinary university based centers, interprofessional team training programs, and workforce development initiatives. These efforts have improved the health and well-being of older adults and contributed to the development of new collaborative care systems. AGHE members have also long been involved in intergenerational teaching and learning strategies, including service-learning and community activities. Intergenerational work in the field of aging has helped to shape the Age-Friendly University initiative which calls for colleges and universities to promote intergenerational learning through reciprocal sharing of expertise among learners of all ages. Finally, the AGHE community has long promoted engaged learning with opportunities for students to interact with community partners through programs such as community-based research and clinical experiences and local policy efforts. The ideas presented in the symposium will lead to reflection on how AGHE can expand its value as GSA’s educational arm.

